# Epigenetic and Structural Brain Aging and Their Associations With Major Depressive Disorder

**DOI:** 10.1016/j.bpsgos.2025.100577

**Published:** 2025-08-05

**Authors:** Eileen Y. Xu, Claire Green, Daniel L. McCartney, Laura K.M. Han, Kathryn L. Evans, Rosie M. Walker, Danni A. Gadd, Douglas Steele, Gordon Waiter, Archie Campbell, Stephen M. Lawrie, James H. Cole, Andrew M. McIntosh, Xueyi Shen, Heather C. Whalley

**Affiliations:** a Division of Psychiatry, Institute for Neuroscience and Cardiovascular Research, University of Edinburgh, Edinburgh, United Kingdom; b Center for Genomic and Experimental Medicine, Institute of Genetics and Cancer, University of Edinburgh, Edinburgh, United Kingdom; c Department of Psychiatry, Amsterdam University Medical Center location Vrije Universiteit Amsterdam, Amsterdam, the Netherlands; d Mood, Anxiety, Psychosis, Sleep & Stress Program, Amsterdam Neuroscience, Amsterdam, the Netherlands; e Amsterdam Public Health, Mental Health program, Amsterdam, the Netherlands; f School of Psychology, University of Exeter, Exeter, United Kingdom; g School of Medicine, University of Dundee, Dundee, United Kingdom; h Aberdeen Biomedical Imaging Centre, School of Medicine, Medical Sciences and Nutrition, University of Aberdeen, Aberdeen, United Kingdom; i Dementia Research Center, Queen Square Institute of Neurology, University College London, London, United Kingdom; j UCL Hawkes Institute, Department of Computer Science, University College London, London, United Kingdom

**Keywords:** Biological age, Brain age, Depression, Epigenetic age, Major depressive disorder, Methylation

## Abstract

**Background:**

A growing body of evidence suggests that major depressive disorder (MDD) may be associated with premature biological aging. However, most studies reported to date have examined brain-based (BrainAge) and DNA methylation (DNAm)–based measures (DNAmAge) of biological age (BioAge) in isolation.

**Methods:**

We investigated 2 well-studied BioAge measures in lifetime and current MDD: BrainAge and DNAmAge (4 separate DNAmAge measures based on Horvath, Hannum, GrimAge, and PhenoAge clocks). We used cross-sectional cohort data from GS:STRADL (Generation Scotland: STratifying Resilience and Depression Longitudinally) (BrainAge *n* = 833; DNAmAge *n* = 587; age range 26–76 years) and used UK Biobank (UKB) data to test for replication of BrainAge associations with MDD (BrainAge *n* = 12,018, age range 45–80 years). Premature brain and DNAm aging were operationalized as predicted age difference (PAD), and analyses controlled for age, sex, smoking, and alcohol intake. We also tested individual and additive associations of brain- and DNAm-based PADs to lifetime/current MDD using logistic regression.

**Results:**

Individuals with lifetime MDD showed significantly higher BrainAge and DNAmAge in GS, ranging from 1.60 to 2.45 years, than individuals without MDD for all measures except for Horvath age. No differences were found for BrainAge in the UKB. In terms of PAD, lifetime MDD was significantly associated with GrimAge-PAD, PhenoAge-PAD, and Brain-PAD, ranging from odds ratio (OR) = 1.21−1.30 (and in UKB, Brain-PAD OR = 1.05). DNAm-PAD and Brain-PAD demonstrated shared and distinctive associations with lifetime MDD, where PhenoAge-PAD plus Brain-PAD explained maximum variance (area under the curve = 0.69, *R*^2^ = 9%). No significant associations were found for current MDD.

**Conclusions:**

Our findings highlight shared and distinct associations of premature brain and DNAm aging in lifetime MDD.

## Plain Language Summary

Depression has been linked to faster biological aging, where people with depression look biologically older than their actual age. Biological aging can be measured in different ways, e.g., chemical changes in DNA across the body or specific changes in the brain seen on brain scans. These have not often been studied together in the same people. We looked at both measures of biological age in the same groups of people with and without depression. Wefound differences in biological aging between people with and without depression, and also found that these body- and brain-based measurements did not fully overlap. This suggests that bodily and brain aging in depression may happen, at least partially, through different biological processes.

Major depressive disorder (MDD) is the leading cause of disability worldwide (1,2). MDD is comorbid with a number of age-related diseases and phenotypes such as type 2 diabetes, cognitive decline, dementia, cardiovascular diseases, and stroke (3). In addition, MDD is associated with an increased risk of mortality, which persists for up to 2 decades after a depressive episode (4). This has informed the theory that MDD may be a state of premature biological aging, where physical and psychological stressors trigger biochemical alterations that lead to age-related molecular changes (5).

Consequently, research has begun to investigate the cellular, molecular, and systemic markers of biological aging in MDD. Early work on biological aging in MDD examined reductions in telomere length—a hallmark of both cellular senescence and organismal aging—in MDD (5). Multiple studies and meta-analyses have found significant negative associations between depression and telomere length, particularly in individuals with more severe depression (6, 7, 8, 9, 10). More recently, work on biological aging in MDD has involved 2 other well-studied estimators of biological age (BioAge) based on DNA methylation (DNAmAge) or on structural brain imaging data (BrainAge) (11, 12, 13, 14, 15, 16).

Taking the former first, DNAm is a chemical modification to DNA where a methyl group is added to a cytosine base in the DNA sequence. This often occurs at CpG sites, where cytosine bases are followed by a guanine base. The methylation status of certain CpG sites can serve as biomarkers for biological processes such as aging, leading to the development of several DNAmAge estimators or epigenetic clocks. These include Hannum, Horvath, GrimAge, and PhenoAge clocks, which use these age-related changes to predict mortality and disease (11, 12, 13, 14,17, 18, 19). Similar to DNAmAge, estimations of BrainAge are based on changes in brain structure over the lifespan; these can also be indicative of poorer brain health and shorter lifespan (15).

Because BioAge estimates (from DNAm or brain imaging data) are closely related to chronological age, these are typically subtracted from, or regressed on, chronological age to derive a biologically informative summary score (the predicted age difference [PAD]). This reflects an individual’s deviation from normal aging trajectories, where a positive PAD indicates a biological age that is greater than chronological age, i.e., premature biological aging. DNAm-PAD, the PAD between individual DNAmAge measures and chronological age, has been shown to be associated with a range of diseases comorbid with MDD, such as cardiovascular diseases, and in smaller studies, with MDD itself (12,18, 19, 20, 21, 22). It is less clear whether DNAm-PADs are directly associated with MDD in larger population studies; however, this may provide insight into mechanisms by which environmental stressors, including early-life adversity, and genetic predispositions may shape gene expression pattern changes associated with MDD.

Regarding BrainAge, MDD is phenotypically and genetically correlated with disorders such as Alzheimer’s disease that have established associations with premature brain aging (12,18, 19, 20, 21). Previous studies have also identified premature brain aging in MDD across adolescence, midlife, and in old age (23, 24, 25). While Brain-PAD (PAD between BrainAge and chronological age) and DNAm-PADs have been independently associated with MDD, combining these measures could boost predictive power and advance our understanding of risk factors (8,26). Concurrent investigation of peripheral and brain-specific PAD measures may also facilitate understanding of shared and distinct mechanisms of premature aging in MDD.

The lack of an integrated investigation into different biological aging markers in MDD is likely due to the lack of available multimodal biological samples collected concurrently in the same cohort. Therefore, in the current study we used a population-based cohort of *n* = 833 unrelated individuals with DNAm and structural magnetic resonance imaging (MRI) data from the same time point, with a BrainAge replication sample of *n* = ∼12,000 participants from the UK Biobank (UKB). First, we tested associations between DNAm-PADs and Brain-PAD in lifetime and current MDD before conducting analysis of Brain-PAD in the replication sample. Then, we tested the unique associations of each of these biomarkers of aging with MDD.

## Methods and Materials

### Generation Scotland

Generation Scotland (GS) is a family- and community-based population cohort comprising ∼20,000 participants recruited from across Scotland between 2006 and 2011 (27). As part of GS:STRADL (STratifying Resilience and Depression Longitudinally), which aimed to investigate the etiology and stratification of depression, a subset of participants were invited for a brain MRI and additional assessment of mental health between 2015 and 2019 (28,29). GS participants in the Tayside and Grampian areas who had a Community Health Index number, were alive and living in Scotland, and had given consent for recontact were invited to take part. Of the 5649 potential participants invited, 646 (11.4%) people declined participation at the first point of contact, and 3358 (59.4%) people did not respond after up to 3 reminders had been sent. Initially, 1645 (29.1%) people responded positively; however, an additional 170 (3.0%) declined once contacted by the research team or withdrew before consenting.

Recruitment ended in May 2019 with 1188 (72.2%) positive respondents having consented to data collection across the Aberdeen and Dundee sites. At each site, participants visited 3 testing stations in a random order for 1) clinical, questionnaire, and biological sample data collection; 2) cognitive assessment; and 3) neuroimaging. After individuals with missing lifetime MDD data were excluded, the final neuroimaging sample consisted of *n* = 833 unrelated participants; *n* = 587 unrelated participants had both neuroimaging and DNAm data (56.7% female; age = 60.5 [9.1] years). Additional details of recruitment and cohort profiling can be found elsewhere (27). Written consent was obtained from all participants. The study was approved by the National Health Service (NHS) Tayside Research Ethics committee (05/s1401/89).

### UK Biobank

The UKB is a prospective cohort study of more than 500,000 individuals from the United Kingdom (30, 31, 32). Participants, ages 40 to 69 years, were invited to one of 22 centers across the UK from 2006 to 2010. The main aims of the resource were to provide a picture of how the health of the UK population develops over many years to enable researchers to improve the diagnosis and treatment of common diseases. In 2014, the UKB reinvited 100,000 participants to undergo brain MRI scanning as well as whole-body imaging. During baseline assessment, extensive sociodemographic, lifestyle, and health-related information was collected together with consent for health data linkage. For further details on recruitment and participant inclusion for imaging assessments, see (30,33).

A subset of UKB neuroimaging data (32), released in 2019, was used in the current study as a replication dataset for BrainAge analysis. This comprised *N* = 12,018 unrelated participants with complete lifetime MDD data (53.3% female; age = 63.2 [7.4] years). Written consent was obtained for all participants. Participants who withdrew up until completion of the study were not included in analyses. Ethical permission was obtained through the NHS Research Ethics Service (11/NW/0382; UKB Project No. 4844).

Travel reimbursement was offered to all participants from both studies. Descriptive statistics for each of these samples are included in Table 1 and Figures S2 and S3.

**Table 1:** Descriptive Statistics for GS:STRADL and the UKB

	GS:STRADL				UKB			
	Lifetime MDD, *n* = 248	No MDD, *n* = 585	Difference	*t*/χ^2^	Lifetime MDD, *n* = 3717	No MDD, *n* = 8301	Difference	*t*/χ^2^
Age, Years	57.64 (9.22)	60.73 (9.55)	−3.09	−4.38∗∗∗	61.52 (7.19)	64.01 (7.33)	−2.49	−17.4∗∗∗
Sex, Female	181 (72.98%)	297 (50.77%)	22.21%	34.2∗∗∗	2527 (67.98%)	3876 (46.69%)	21.29%	467∗∗∗
Smoking, Ever Smoker	123 (49.50%)	226 (38.63%)	10.87%	7.04∗∗	1449 (38.98%)	2866 (34.53%)	4.45%	23.3∗∗∗
Alcohol Units per Week	7.04 (10.42)	8.05 (9.28)	−1.01	−1.32	11.45 (13.17)	13.50 (13.69)	−2.05	−7.7∗∗∗
BMI	28.67 (5.46)	27.39 (5.09)	1.28	3.15∗∗	27.04 (4.88)	26.14 (4.09)	0.9	−9.63∗∗∗
Current MDD Status	38 (15.32%)	–			641 (17.24%)	–		
BrainAge	58.84 (13.47)	56.41 (11.50)	2.43	2.48∗	62.76 (9.96)	62.52 (10.13)	0.25	1.25
Horvath Age	61.59 (7.56)	60.71 (7.91)	0.88	1.26	–	–	–	–
Hannum Age	52.34 (8.44)	50.74 (8.88)	1.60	2.06∗	–	–	–	–
PhenoAge	51.52 (8.90)	49.17 (9.47)	2.35	2.85∗∗	–	–	–	–
GrimAge	61.84 (8.03)	59.80 (8.27)	2.04	2.77∗∗	–	–	–	–

Values are presented as mean (SD) or *n* (%).

∗*p* < .05, ∗∗*p* < .01, ∗∗∗*p* < .001—indicate statistically significant differences between individuals with and without lifetime MDD.

BMI, body mass index; GS:STRADL, Generation Scotland: STratifying Resilience and Depression Longitudinally; MDD, major depressive disorder; UKB, UK Biobank.

### Definition of MDD

Participants in GS:STRADL were assessed for mood disorder symptoms (MDD, mania, and hypomania) using the Structured Clinical Interview for DSM-IV Disorders (SCID), Research Version (34). SCID diagnostic criteria are consistent with symptom criteria for unipolar depression and bipolar disorder in DSM-IV (29). Responses were evaluated against MDD criteria, and participants were classified as individuals with or without lifetime MDD based on whether they had experienced at least 1 major depressive episode by the time of assessment. Individuals with lifetime MDD were then evaluated for current MDD based on the SCID and DSM-IV criteria for MDD.

In the UKB, individuals with and without lifetime MDD were identified based on the self-report Composite International Diagnostic Interview–Short Form, administered by online questionnaire after the baseline assessment (35). Individuals with current MDD were classified based on the 9-item Patient Health Questionnaire (PHQ-9), which assesses MDD symptoms during the preceding 2 weeks (36,37).

### DNAm-PAD

DNAm data from GS:STRADL were profiled in 2 batches from whole blood samples using the Illumina Infinium MethylationEPIC BeadChip (Illumina Inc.) according to the manufacturer’s protocol. Data preprocessing was performed consistent with methods used in the baseline GS sample (38), although the methylation-derived smoking score described by Walker *et. al.* (38) was not available at the time and is not included in this article. Quality control and preprocessing were performed separately for each batch in R 3.6.1 (39) using minfi (40) and wateRmelon (41); samples were removed where >0.5% of CpGs had a detection *p* value > .01, probes where >1% of samples had a detection *p* value > .01, and probes with a bead count of <3 in >5% of samples. Data were background corrected using normal-exponential out-of-band preprocessing (42). Finally, reported biological sex assigned at birth was confirmed by DNAm data; no other exclusions were made.

Raw DNAmAge estimates were calculated from GS:STRADL DNAm data using the DNA Methylation Age Calculator (http://dnamage.genetics.ucla.edu/) (14,43). A total of 4 measures were generated: Horvath age, Hannum age, PhenoAge, and GrimAge (11,13,14,17). More details on each DNAmAge measure are presented in the Supplement.

DNAm-PADs were defined as the residual term from regressing each raw DNAmAge estimate against chronological age, including sex, batch, and DNAm-estimated cell proportions taken at blood draw as covariates. Following this, we rescaled DNAm-PAD residuals to the raw DNAmAge mean and SD for each DNAmAge measure to generate an age-adjusted estimate of DNAmAge with the effects of chronological age and covariates removed, in units of years. Throughout the article, we refer to estimates of DNAmAge and DNAm-PAD as per the naming of originating clocks (e.g., Hannum age, Hannum-PAD, GrimAge, GrimAge-PAD), where a positive DNAm-PAD indicates accelerated or premature aging, and a negative DNAm-PAD indicates decelerated or delayed aging.

### Brain-PAD

GS:STRADL MRI data were acquired and preprocessed using a unified protocol (29). Data were acquired at 2 sites using a 3T Philips Achieva TX scanner and 32-channel head coil at one site and a 3T Siemens Prisma-FIT scanner and 20-channel head coil at the second site. T1-weighted images from the UKB (released in 2019) were acquired at 2 sites, each of which used a 3T Siemens Skyra scanner and 32-channel head coil by the UKB imaging team (32). Further details have been published previously for both GS:STRADL (29) and the UKB (32).

Raw BrainAge estimates were predicted using the brainageR software version 2.1 (https://github.com/james-cole/brainageR), which included SPM12 (https://www.fil.ion.ucl.ac.uk/spm/doc/) and FSL preprocessing (15). brainageR uses principal components derived from voxelwise volumetric data in normalized gray matter, white matter, and cerebrospinal fluid segmentations to predict age. The brainageR model was trained on 3377 healthy participants, ages 18 to 92 years, from 7 sites (15).

Similar to DNAm-PAD, Brain-PAD was defined as the residual term from regressing raw BrainAge against chronological age with sex and scan site as covariates. In the UKB, scanner head position coordinates were also included to adjust for potential bias caused by uneven static MRI field. Following this, Brain-PAD residuals were rescaled to the raw BrainAge mean and SD to generate a new estimate of BrainAge in years minus the effects of chronological age. Throughout the article, BrainAge refers to this age-adjusted estimate in years, and Brain-PAD refers to the age difference relative to chronological age; positive Brain-PAD indicates accelerated or premature brain aging, and negative Brain-PAD indicates delayed brain aging.

### BioAge Measures

All 5 raw BioAge estimations (4 DNAmAge estimates and BrainAge) were compared against chronological age using Pearson’s correlations. MDD-related differences in mean age-adjusted BioAge were compared using *t* tests.

### MDD-Related Differences in BioAge-PAD

Associations between MDD status and BioAge-PAD, the PAD between biological age and chronological age, were tested using logistic regression models. Participant age and sex were included as covariates together with lifetime smoking (binary ever/never) and alcohol consumption (self-reported units per week) because these lifestyle factors are known to be associated with BioAge and MDD (18, 19, 20,25). Where there was a significant interaction between BrainAge and assessment center, this interaction term was added as an additional covariate in Brain-PAD models. Effect sizes are reported as standardized log-transformed odds ratios; values >1 indicate increased aging in individuals with MDD compared with individuals without MDD.

### Statistical Analysis

Statistical analyses were performed in R version 4.1.0 and 4.1.3 (39). In addition to the modality-specific covariates described, models that assessed associations with MDD outcomes controlled for age, sex, smoking (ever smoker; self-reported binary variable), and alcohol use (in self-reported units per week). A sensitivity analysis that also includes neighborhood deprivation, recent life events, and childhood adversity covariates is presented in the Supplement. Model performance and comparison metrics were generated using the R package performance version 0.10.4 (44). In GS:STRADL analyses, *p* values were false discovery rate (FDR) adjusted across the 5 BioAge measures. *p* Values were not adjusted for multiple comparisons in the UKB because there was only 1 BioAge measure (i.e., BrainAge). In all analyses, statistical significance was determined using a cutoff of *p*_FDR_ < .05 for adjusted analyses and α < 0.05 for unadjusted analyses.

### Additive Associations of DNAm-PAD and Brain-PAD

Additive associations of DNAm-PAD and Brain-PAD were assessed by stepwise comparison of models adjusted for age and sex only. One null model and 2 sets of testing models were created to compare the addition of Brain-PAD to DNAm-PAD–only models for each DNAm-PAD measure (i.e., tested individually) and vice versa:

1. H1 model: lifetime MDD ∼ age + sex
2. H2 models: lifetime MDD ∼ age + sex + DNAm-PAD or Brain-PAD
3. H3 model: lifetime MDD ∼ age + sex + DNAm-PAD and Brain-PAD.

H2 models were compared against the H1 model to evaluate the variance explained in MDD by each individual DNAm-PAD or Brain-PAD. The H3 model was then compared against each of the H2 models to obtain the increased variance explained by including DNAm-PAD and Brain-PAD together.

The area under the curve (AUC) and Tjur’s *R*^2^ were compared for each set of models to quantify improvements in model fit. Tjur’s *R*^2^ was selected over other pseudo-*R*^2^s due to its similarity with *R*^2^ for linear models (45); Tjur’s *R*^2^ values closer to 1 indicate greater separation between predicted probabilities for individuals with MDD and individuals without MDD. Model comparisons were made using χ^2^ tests, and a heatmap of correlations between BioAge-PAD measures is presented in Figure S5.

## Results

Sample descriptions are reported in Table 1. Participant ages were similar in the 2 samples. Notably, the number of individuals with current MDD was relatively small, with *n* = 38 in GS:STRADL (15% of lifetime MDD) and *n* = 641 in the UKB (17% of lifetime MDD).

### BioAge Measures

Raw BioAge estimates were highly correlated with chronological age across all DNAmAges (Figure S1). Hannum age showed the strongest correlation (Pearson’s *r* = 0.90, *p* < .001), followed by Horvath age (*r* = 0.88, *p* < .001), GrimAge (*r* = 0.86, *p* < .001), and PhenoAge (*r* = 0.83, *p* < .01). The accuracy of raw BrainAge predictions in terms of correlation with chronological age were *r* = 0.82 (*p* < .001) for GS:STRADL and *r* = 0.79 (*p* < .001) for the UKB.

Individuals with lifetime and current MDD had higher mean BioAges than individuals without MDD (Table 1 and Figure S4). For lifetime MDD, the greatest mean difference was for BrainAge (2.43 years), followed by PhenoAge (2.35 years), GrimAge (2.04 years), and Hannum age (1.60 years); the smallest difference was for Horvath age (0.88 years). Differences were statistically significant for all except Horvath age.

For current MDD, GrimAge showed the greatest difference compared with individuals without MDD (4.69 years), followed by PhenoAge (4.27 years), Hannum age (3.96 years), BrainAge (2.79 years), and Horvath age (2.20 years). Differences were statistically significant for GrimAge and PhenoAge only.

Smaller differences were found for BrainAge in the UKB for lifetime MDD (0.25 years) and current MDD (0.60 years); these were not statistically significant.

### MDD and BioAge-PAD

#### Lifetime MDD

**Figure 1:**
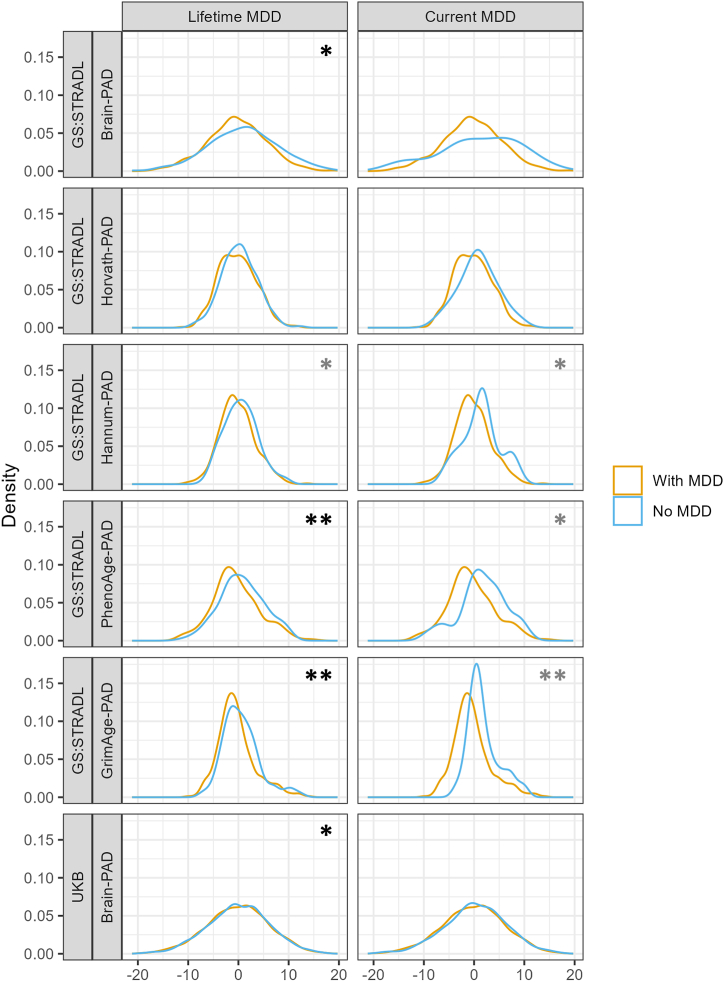
Distribution of BioAge-PADs in GS:STRADL and the UK Biobank. Distribution of BioAge-PAD measures across individuals with lifetime and current MDD and individuals without MDD. Gray asterisks represent statistically significant associations with MDD status prior to FDR correction*;* black asterisks represent statistical significance after FDR correction. ∗*p* < .05, ∗∗*p* < .01. GS:STRADL, Generation Scotland: STratifying Resilience and Depression Longitudinally; FDR, false discovery rate; MDD, major depressive disorder; PAD, predicted age difference; UKB, UK Biobank.

Following FDR correction for multiple testing, PhenoAge-PAD (OR = 1.30; 95% CI, 1.07–1.57; *p*_FDR_ = .03), GrimAge-PAD (OR = 1.28; 95% CI, 1.04–1.57; *p*_FDR_ = .04), and Brain-PAD (OR = 1.21; 95% CI, 1.03–1.42; *p*_FDR_ = .04) were associated with increased odds of lifetime MDD. The association between Brain-PAD and MDD was replicated in the UKB, although the increase in odds per 1 SD increase was smaller (OR = 1.05; 95% CI, 1.003–1.10; *p* = .04) (Figure 1).

#### Current MDD

Prior to FDR correction, a 1 SD increase in Hannum-PAD (OR = 1.51; 95% CI, 1.02–2.25; *p* = .04) and GrimAge-PAD (OR = 1.76; 95% CI, 1.14–2.73; *p* = .01) were associated with increased odds of current MDD. However, none of these associations survived FDR correction. No significant associations were found for Brain-PAD with current MDD in either GS:STRADL or the UKB (Figure 1).

### Additive Associations of DNAm-PAD and Brain-PAD

Comparisons were performed to test the combined associations of DNAm-PAD and Brain-PAD over and above a single measure only (DNAm-PAD or Brain-PAD alone) when predicting MDD status.

**Figure 2:**
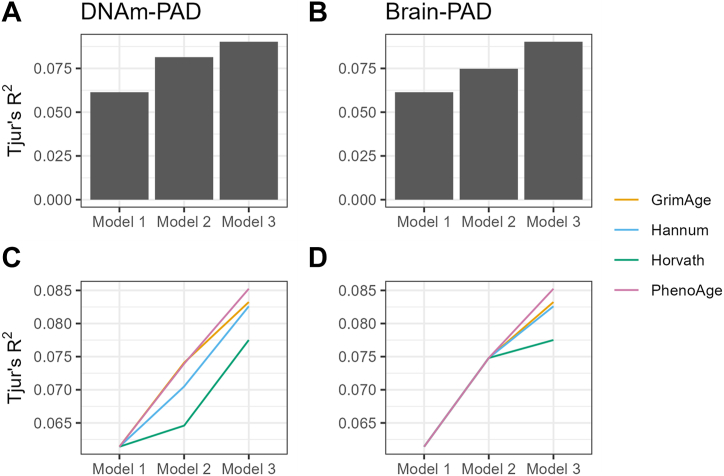
Combined models of Brain-PAD and DNAm-PAD. Comparisons of *R*^2^ across models of lifetime MDD in GS:STRADL. In panels **(A)** and **(B)**, all 4 DNAm-PAD measures are added simultaneously (Horvath-PAD, Hannum-PAD, PhenoAge-PAD*,* and GrimAge-PAD); panels **(C)** and **(D)** present the increase in *R*^2^ for each DNAm-PAD measure individually when combined with Brain-PAD. Model 1: Lifetime MDD predicted by age and sex only. Model 2: Lifetime MDD predicted by age, sex*,* and DNAm-PAD **(A)** or Brain-PAD **(B)**. Model 3: Lifetime MDD predicted by age, sex, DNAm-PAD, and Brain-PAD. DNAm, DNA methylation; GS:STRADL, Generation Scotland: STratifying Resilience and Depression Longitudinally; MDD, major depressive disorder; PAD, predicted age difference.

For lifetime MDD, the model with greatest *R*^2^ was for PhenoAge-PAD and Brain-PAD combined (AUC = 0.69, *R*^2^ = 9%) (Figure 2B, C). The addition of Brain-PAD to DNAm-PAD models significantly improved fit for Horvath-PAD (χ^2^_1_ = 5.77, *p* = .02, ΔAUC = 0.012, Δ*R*^2^ = 0.012), Hannum-PAD (χ^2^_1_ = 5.13, *p* = .02, ΔAUC = 0.013, Δ*R*^2^ = 0.011), PhenoAge-PAD (χ^2^_1_ = 4.43, *p* = .04, ΔAUC = 0.011, Δ*R*^2^ = 0.009), and GrimAge-PAD (χ^2^_1_ = 4.07, *p* = .04, ΔAUC = 0.008, Δ*R*^2^ = 0.009) (Figure 2C). The reverse analyses of adding DNAm-PAD to Brain-PAD models also resulted in significant improvement in model fit, with the exception of Horvath-PAD (Hannum-PAD: χ^2^_1_ = 3.99, *p* = .05, ΔAUC = 0.007, Δ*R*^2^ = 0.006; PhenoAge-PAD: χ^2^_1_ = 7.50, *p* = .006, ΔAUC = 0.014, Δ*R*^2^ = 0.010; GrimAge-PAD: χ^2^_1_ = 5.63, *p* = .02, ΔAUC = 0.001, Δ*R*^2^ = 0.008) (Figure 2D). When all DNAm-PAD measures were combined, the addition of Brain-PAD slightly improved model fit (χ^2^_1_ = 4.00, *p* = .045, ΔAUC = 0.008, Δ*R*^2^ = 0.009) (Figure 2A). Similarly, adding all DNAm-PAD measures simultaneously to Brain-PAD also resulted in improved fit (χ^2^_4_ = 10.93, *p* = .027, ΔAUC = 0.020, Δ*R*^2^ = 0.015) (Figure 2B).

## Discussion

In this study, we explored 1) the relationship between markers of biological aging and MDD using different tissue types (DNAm and brain) and 2) their individual and combined associations with MDD. These analyses were conducted in a relatively large population-based sample of unrelated individuals, with replication of brain age findings in an independent sample. We focused on 2 different but complementary types of biological aging, blood-based DNAmAge and BrainAge. We also focused on 2 main MDD phenotypes, lifetime and current MDD.

We found significant associations between lifetime MDD and PhenoAge-PAD, GrimAge-PAD, and Brain-PAD. The finding of increased brain aging was further validated in a large, independent sample from the UKB. This provides further demonstration of premature brain aging in MDD across cohorts utilizing high-dimensional imaging data, which adds to the literature on findings based on derived imaging phenotypes. Effect sizes of associations with MDD were comparable between Brain-PAD and DNAm-PADs in GS:STRADL, with a 1 SD increase in each BioAge-PAD associated with a 20% to 30% increase in odds of lifetime MDD. This was less pronounced in the UKB, where a 1 SD increase in Brain-PAD was associated with a 5% increase in odds. We also report that a combination of blood- and brain-based measures of aging significantly improved classification of individuals with MDD and without MDD versus either measure in isolation, indicating separate and combined associations of aging from DNAm-PAD and Brain-PAD measures. In particular, the combination of PhenoAge-PAD and Brain-PAD explained most variance associated with MDD. Therefore, this study provides evidence of premature biological aging in MDD, extending current literature through replication of increased brain aging in MDD and bringing together different types of biological aging, thereby demonstrating the importance of both peripheral and central biological aging processes in MDD.

Previous studies have typically investigated peripheral and neurobiological measures of biological aging separately in MDD. Results for second-generation DNAm clocks included in the current study—PhenoAge and GrimAge—have largely been mixed to date. Some studies have reported positive associations between DNAmAge and MDD/depressive symptoms (22,46, 47, 48, 49) or no associations (50,51), with positive associations primarily reported for GrimAge (22,46,47). Only 2 previous studies have reported significant associations with PhenoAge in depression—one twin study that reported associations between PhenoAge and continuous depression scores (52) and another study that examined relationships between childhood adversity and increased biological aging in individuals with depression (53). In terms of their derivation, GrimAge incorporates epigenetic markers of smoking, whereas PhenoAge incorporates phenotypic markers of aging including physiological status and morbidity profiles. Therefore, our results may point to additional physiological aging processes in MDD that are captured by PhenoAge measures. Regarding BrainAge, a recent meta-analysis reported a small increase in Brain-PAD in MDD (of +0.90 [0.20] years), with some variation based on whether Brain-PAD was calculated based on derived imaging metrics, as in ENIGMA (Enhancing Neuro Imaging Genetics through Meta Analysis) mega-analysis studies, or based on the original high-dimensional imaging data, as in the current study (54). In sum, by leveraging concurrent high-dimensional neuroimaging and methylation data, our results point to premature biological aging across both the brain and periphery in MDD. We found separate and combined associations of peripheral and central markers of aging that were more predictive of MDD when combined than for either measure in isolation.

After also adjusting for the effects of childhood trauma, recent life events, and socioeconomic status, we found no significant associations between BioAge-PAD and MDD (see the Supplement). This null result may represent insufficient statistical power due to a reduced sample size; however, it may be that these factors are relevant in the bidirectional relationships between aging and MDD (10,55). Further studies that concurrently examine these temporal adversity measures are required to draw firmer conclusions. One commonly proposed biological mechanism that may connect aging and MDD is inflammation, which has consistently been associated individually with both premature aging (56) and MDD pathogenesis (57,58). As the extent to which acute and chronic inflammation contributes to the relationship between MDD and premature biological aging remains uncertain, future work could explore the contribution of acute and stable markers of inflammation to premature biological aging associated with MDD (57).

It is important to note as a limitation of the current study that the ethnic backgrounds of our samples were highly homogeneous (mainly northern European), which limits the generalizability of our findings to other ethnic groups. We also note that the current study was cross-sectional, and we did not examine directions of causality. This work could be expanded on by using existing Mendelian randomization studies that indicate bidirectional relationships between aging and MDD (10,56) to include different types of biological aging to gain a deeper understanding of its causal relationship with MDD. Harmonizing assessment tools for depression across cohorts may also compromise statistical power for replication; however, previous work has shown acceptable transferability between the 2 scales used in the GS and UKB (SCID and PHQ-9, respectively) (57), with the proportion of cases accurately predicted by PHQ-9 being 73.80%. Finally, we highlight that the observed associations should be interpreted in light of potential selection biases. Individuals who contributed biomarker or imaging data in both studies have been reported to differ in health and sociodemographic factors from the broader populations from which each cohort was derived (28,58). Therefore, our findings may reflect characteristics of a more engaged or health-conscious subset of individuals, which could limit generalizability and potentially influence effect estimates.

### Conclusions

Our findings demonstrate that premature aging in MDD is observed across both brain and peripheral measures of biological aging, with evidence of shared and distinct associations of each. Suggestions are made for future work to explore potential biological mechanisms in greater detail.

## Supplementary Material

Supplemental Text, Figures S1–S5, and Table S1

Key Resources Table
