# Supplementary material for: Epigenetic and Structural Brain Aging and Their Associations With Major Depressive Disorder
Source: Biol Psychiatry Glob Open Sci. 2025 Aug 5;5(6):100577. doi: 10.1016/j.bpsgos.2025.100577 (PMC12454886; doi:10.1016/j.bpsgos.2025.100577)
Supplement: Supplemental Text, Figures S1–S5, and Table S1 [file mmc1.pdf]

SUPPLEMENTARY INFORMATION

Epigenetic and Structural Brain Aging and Their Associations With Major Depressive Disorder

Xu *et al.*

Contents

DNAmAge measures 2
Sensitivity analysis with additional covariates 3
Table S1: SIMD, Childhood Trauma and Recent Life Events in GS:STRADL 4
Figure S1: Correlation between BioAge estimates and chronological age 5
Figure S2: GS:STRADL demographics by MDD phenotype 6
Figure S3: UK Biobank demographics by MDD phenotypes 7
Figure S4: Differences in BioAge by MDD phenotype 8
Figure S5: Correlation between BioAge-PAD measures in GS:STRADL 9
References: 10

DNAmAge measures

The “first-generation” DNAm clocks, developed by Horvath (1) and Hannum *et al.* (2) were trained on chronological age. Acceleration in DNAm Horvath and Hannum age – a DNAm age greater than chronological age – moderately predicted age-related morbidity (3, 4). In contrast, “second-generation” clocks DNAmPhenoAge and DNAmGrimAge were developed using a two-stage approach (5, 6). These clocks were trained using a combination of clinical markers that associated with physical functioning and mortality (DNAmPhenoAge) and time-to-death (DNAmGrimAge). These included white blood cell count and C-reactive protein (for DNAmPhenoAge) and DNAm surrogates of leptin, adrenomedullin and smoking pack-years (for DNAmGrimAge). In comparison to first-generation clocks, DNAmPhenoAge and DNAmGrimAge accelerations show stronger associations with mortality and time-to-death (5-7). Details for each DNAm Age are listed below:

- *Horvath age:* An ‘epigenetic clock’ predicting age from DNA methylation at 353 CpG sites (1). During development, the model was trained on chronological age using methylation data from 8,000 samples taken from a range of tissues.
- *Hannum age:* An alternative epigenetic clock, also developed by training a model on chronological age and DNA methylation data. The training dataset for this model included more than 450,000 CpG sites, and 656 samples all taken from blood (2).
- *DNAmPhenoAge:* A more elaborate epigenetic clock trained not only on chronological age, but also on selected clinical factors associated with ageing-related mortality, such as cardiovascular disease, Alzheimer’s disease, diabetes and chronic lower respiratory disease (5). This model predicts age from methylation at 513 CpG sites and was trained using the NHANES dataset.
- *DNAmGrimAge:* An epigenetic clock predicting mortality rather than age, trained on DNA methylation but also seven related plasma protein levels (6). Although focused on time to death rather than current biological age, the online calculator still provided a methylation age estimate based on this model.

Sensitivity analysis with additional covariates

To adjust for other proximal and distal factors associated with MDD, three additional covariates were added for analyses in GS:STRADL only. These were quintiles from the Scottish Index of Multiple Deprivation (SIMD) (8) for the individual's current address, Childhood Trauma Questionnaire (CTQ) score – indicating the extent and severity of childhood trauma exposure (9) – and total number of stressful life events in the past six months, assessed by the 12-item Brief Life Events Questionnaire (BLEQ). After excluding individuals with missing covariate data, sensitivity analysis for Brain-PAD was conducted in N=660 individuals; sensitivity analyses for DNAm-PADs were conducted in N=~460 individuals. A summary of these measures by lifetime MDD status are provided in Table S1. The resulting models adjust for: age, sex, lifetime smoking, alcohol consumption (units/week), SIMD quintile, CTQ score and number of recent life events.

Prior to FDR correction for multiple testing, PhenoAge-PAD was associated with increased odds of lifetime MDD (OR = 1.29; 95% CI: 1.03 – 1.63; p = .03). However, this association did not survive FDR correction (adjusted p = .15). No significant associations were found between lifetime MDD and Brain-PAD, Horvath-PAD, Hannum-PAD or GrimAge-PAD after inclusion of these additional covariates. Similarly, no significant associations were found between any BioAge-PAD measures and current MDD.

Table S1: SIMD, Childhood Trauma and Recent Life Events in GS:STRADL

	Lifetime MDD (Total N = 248)	No MDD (Total N = 585)	t/χ^2
SIMD Quintile [n (%)]			14.02 **
1 (most deprived)	17 (8.29%)	13 (2.83%)	
2	27 (13.17%)	45 (9.78%)	
3	41 (20%)	88 (19.13%)	
4	42 (20.49%)	127 (27.61%)	
5 (least deprived)	78 (38.05%)	187 (40.65%)	
Missing	43 (17.34%)	125 (21.37%)	
BLEQ [mean (SD)]	1.22 (1.6)	0.77 (1.15)	-3.70 ***
Missing [n (%)]	36 (14.52%)	106 (18.12%)	
CTQ [mean (SD)]	38.31 (15.02)	31.18 (8.29)	-6.49 ***
Missing [n (%)]	36 (14.52%)	106 (18.12%)	

Table S1: Description of additional covariates included in GS:STRADL sensitivity analyses for individuals with and without lifetime MDD. GS:STRADL = Generation Scotland: Stratifying Resilience and Depression Longitudinally. MDD = Major Depressive Disorder. SIMD = Scottish Index of Multiple Deprivation. BLEQ = Brief Life Events Questionnaire. CTQ = Childhood Trauma Questionnaire. *, ** and *** represent significant differences between individuals with MDD and individuals without MDD at $p<.05$, $p<.01$ and $p<.001$, respectively.

Figure S1: Correlation between BioAge estimates and chronological age

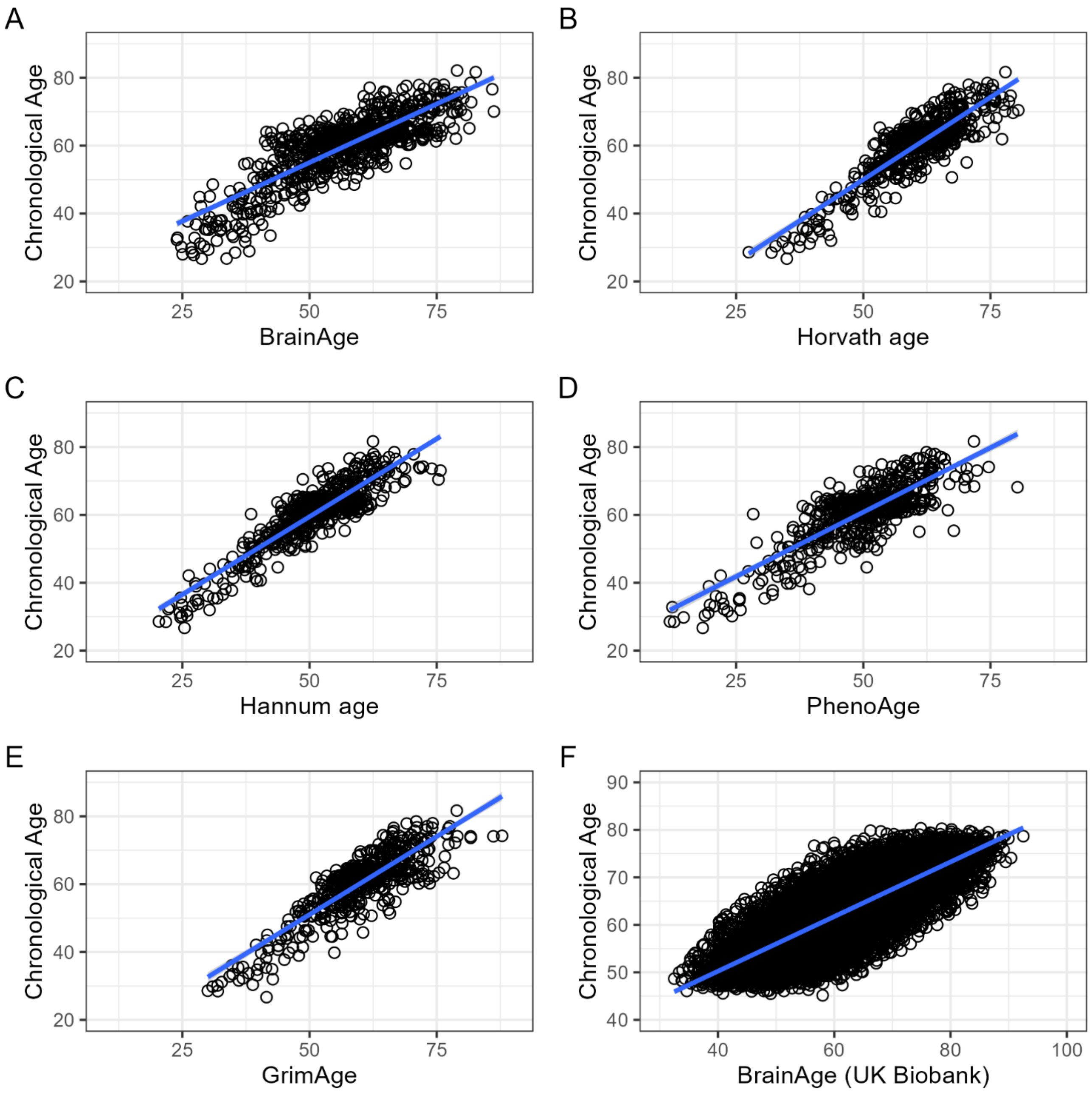

Figure S1: DNAmAge and BrainAge estimates plotted against chronological age in GS:STRADL (A-E) and UK Biobank (F). GS:STRADL = Generation Scotland: Stratifying Resilience and Depression Longitudinally.

Figure S2: GS:STRADL demographics by MDD phenotype

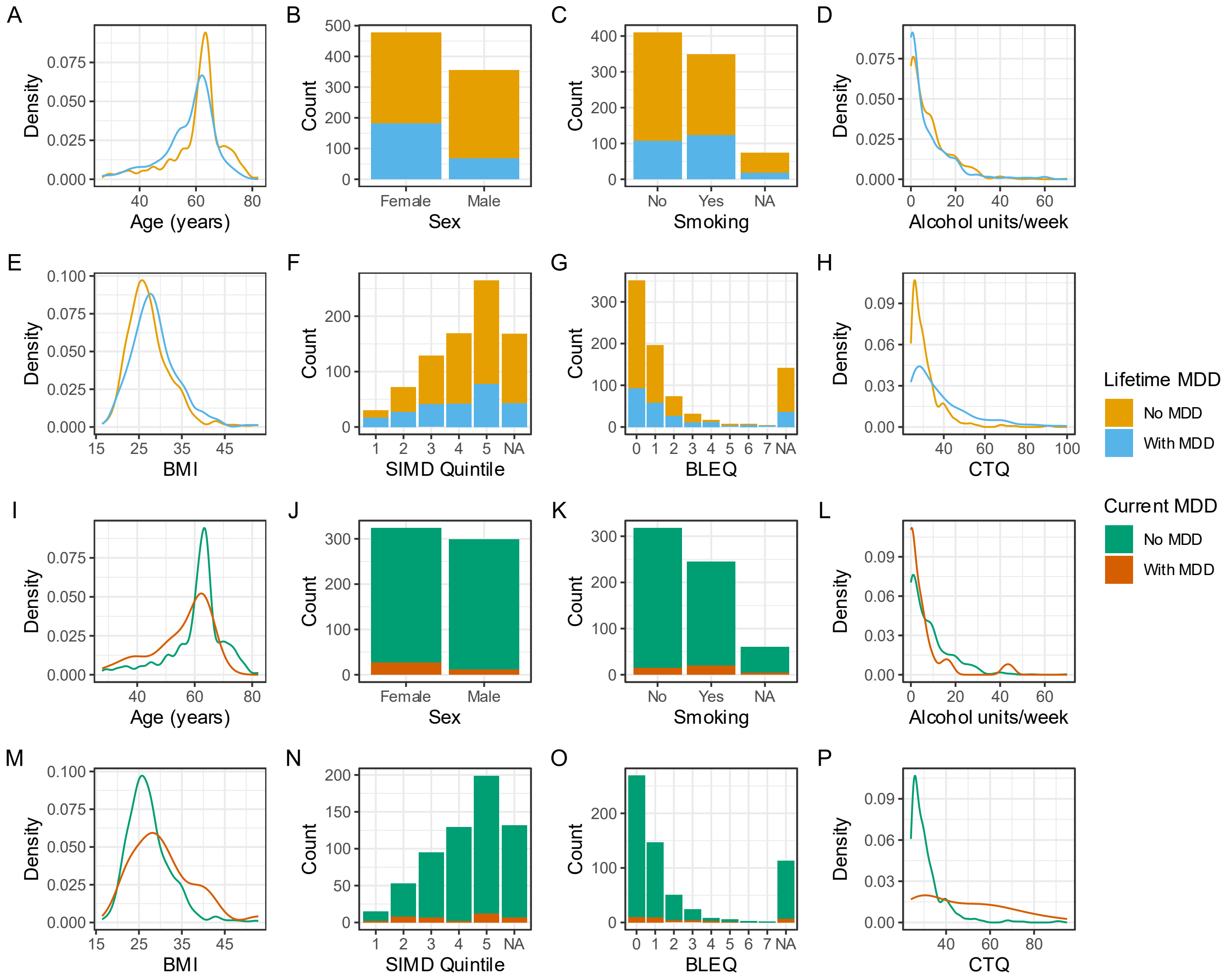

Figure S2: Demographic information for lifetime MDD (A-H) and current MDD (I-P) in GS:STRADL. BMI = body mass index. SIMD = Scottish Index of Multiple Deprivation. BLEQ = Brief Life Events Questionnaire. CTQ = Childhood Trauma Questionnaire. GS:STRADL = Generation Scotland: Stratifying Resilience and Depression Longitudinally. MDD = Major Depressive Disorder.

Figure S3: UK Biobank demographics by MDD phenotypes

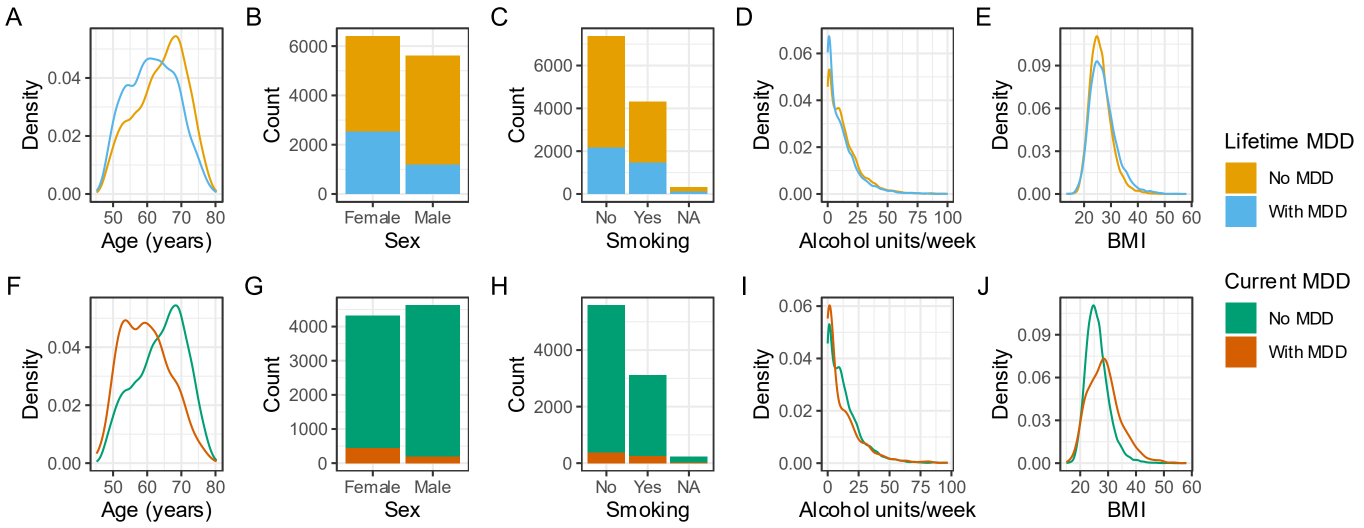

Figure S3: Demographic information for lifetime MDD (A-E) and current MDD (F-J) in UK Biobank. BMI = body mass index. MDD = Major Depressive Disorder.

Figure S4: Differences in BioAge by MDD phenotype

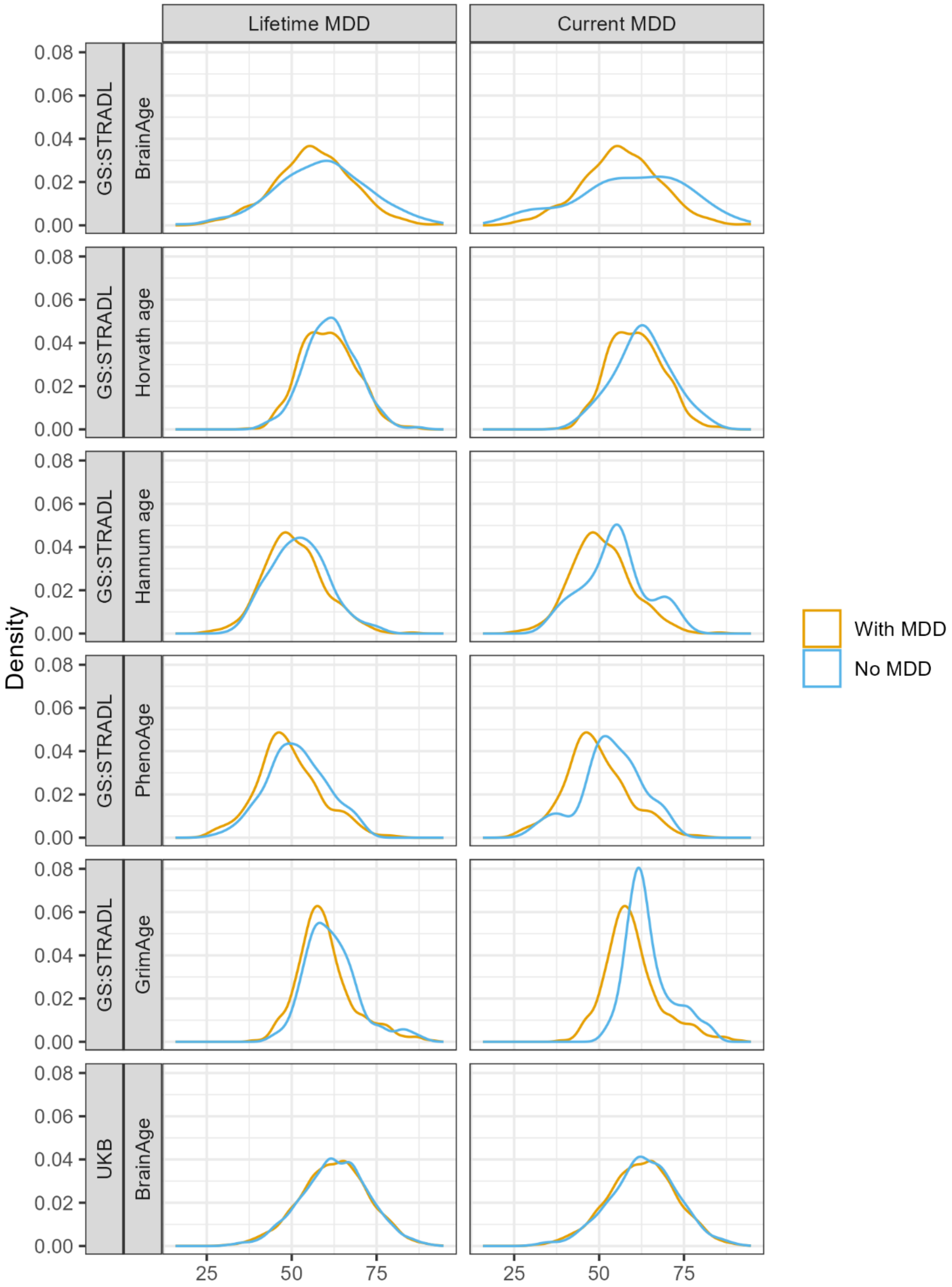

Figure S4: Distributions of BrainAge and DNAmAge estimates in GS:STRADL and UK Biobank. GS:STRADL = Generation Scotland: Stratifying Resilience and Depression Longitudinally. UKB = UK Biobank. MDD = Major Depressive Disorder.

Figure S5: Correlation between BioAge-PAD measures in GS:STRADL

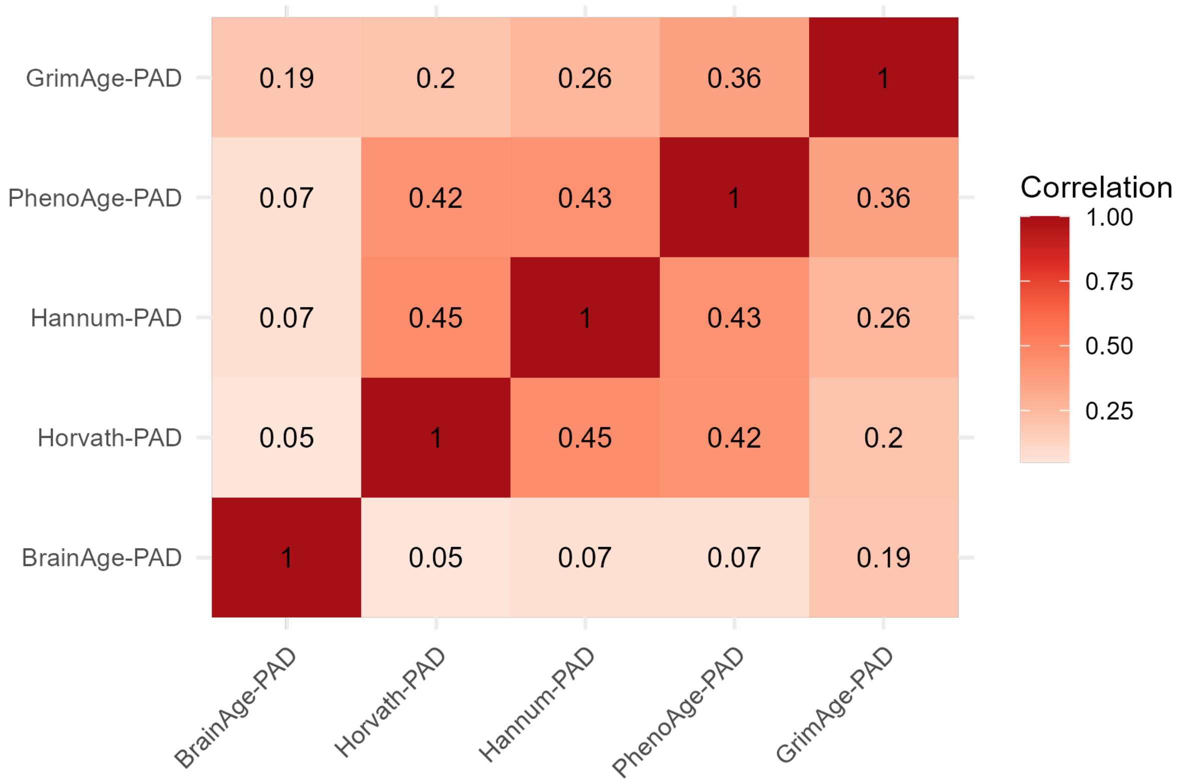

Figure S5: Heatmap of Pearson correlations between Brain-PAD and DNAm-PAD measures. PAD = Predicted Age Difference. GS:STRADL = Generation Scotland: Stratifying Resilience and Depression Longitudinally.

References:

1. Horvath S (2013): DNA methylation age of human tissues and cell types. *Genome Biology*. 14:3156.

2. Hannum G, Guinney J, Zhao L, Zhang L, Hughes G, Sadda S, et al. (2013): Genome-wide methylation profiles reveal quantitative views of human aging rates. *Mol Cell*. 49:359-367.

3. Quach A, Levine ME, Tanaka T, Lu AT, Chen BH, Ferrucci L, et al. (2017): Epigenetic clock analysis of diet, exercise, education, and lifestyle factors. *Aging (Albany NY)*. 9:419-446.

4. Horvath S, Raj K (2018): DNA methylation-based biomarkers and the epigenetic clock theory of ageing. *Nature Reviews Genetics*. 19:371-384.

5. Levine ME, Lu AT, Quach A, Chen BH, Assimes TL, Bandinelli S, et al. (2018): An epigenetic biomarker of aging for lifespan and healthspan. *Aging (Albany NY)*. 10:573-591.

6. Lu AT, Quach A, Wilson JG, Reiner AP, Aviv A, Raj K, et al. (2019): DNA methylation GrimAge strongly predicts lifespan and healthspan. *Aging*. 11:303-327.

7. Oblak L, van der Zaag J, Higgins-Chen AT, Levine ME, Boks MP (2021): A systematic review of biological, social and environmental factors associated with epigenetic clock acceleration. *Ageing Research Reviews*. 69:101348.

8. Scottish Government (2020): Scottish Index of Multiple Deprivation 2020.

9. Hagborg JM, Kalin T, Gerdner A (2022): The Childhood Trauma Questionnaire-Short Form (CTQ-SF) used with adolescents - methodological report from clinical and community samples. *J Child Adolesc Trauma*. 15:1199-1213.
